# Identification and clinical validation of F3 as a peripheral blood biomarker for neonatal hypoxic-ischemic encephalopathy using dataset-specific bioinformatics analysis

**DOI:** 10.3389/fped.2026.1891438

**Published:** 2026-08-25

**Authors:** Suxiang Pan, Yanan Peng, Jianqing Hu, Yanping Cai, Yibing Zhang, Bijuan Zheng

**Affiliations:** 1 Department of Neonatal Intensive Care Unit, Affiliated Hospital of Putian University, Putian, China; 2 Department of Pediatrics, First People’s Hospital of Wuhu, Wuhu, China

**Keywords:** bioinformatics, biomarker, clinical validation., f3, neonatal hypoxic-ischemic encephalopathy (HIE), tissue factor, transcriptomics

## Abstract

**Background:**

Neonatal hypoxic-ischemic encephalopathy (HIE) is a major cause of neonatal death and long-term neurological disability. Early recognition of high-risk neonates remains difficult because clinical examination, imaging, electrophysiology, and biochemical tests may be limited by timing, access, or early sensitivity. We aimed to identify and clinically validate a peripheral blood biomarker candidate for HIE.

**Methods:**

Public transcriptomic datasets related to neonatal HIE or experimental hypoxic-ischemic injury were analyzed as separate biological resources. The human whole-blood dataset served as the primary discovery dataset. Experimental rat cortex datasets were analyzed separately and used only as supportive context after ortholog mapping. Candidate prioritization considered nominal human blood evidence, directionally concordant supportive transcriptomic signals, HIE-related disease-gene annotation, and validation in human peripheral blood mononuclear cells (PBMCs). F3 mRNA was measured by qRT-PCR, and full-length tissue factor (flTF) protein was assessed by immunofluorescence and Western blotting in an independent cohort of neonates with HIE (*n* = 40) and non-HIE controls (*n* = 30).

**Results:**

With an exploratory nominal screening threshold, dataset-specific analysis identified F3 as a candidate gene with nominal upregulation in the human whole-blood dataset (log2FC = 6.27, nominal *P* = 0.041, adjusted *P* = 0.524). Independent experimental hypoxic-ischemic injury datasets showed directionally concordant supportive signals. In the clinical cohort, PBMC F3 mRNA was higher in neonates with HIE than in non-HIE controls (4.2 ± 0.2 vs. 1.0 ± 0.1, *P* < 0.0001), with increased flTF protein expression. ROC analysis showed good discrimination in this cohort (AUC, 0.844; 95% CI, 0.735–0.953).

**Conclusion:**

F3/flTF was prioritized as an exploratory peripheral blood biomarker candidate on the basis of nominal human whole-blood transcriptomic evidence, supportive disease-model data, HIE-related disease-gene annotation, and independent PBMC validation. PBMC F3/flTF may help identify HIE and stratify risk, but larger human blood cohorts, confounder-adjusted analyses, and mechanistic studies are needed before clinical use can be considered.

## Introduction

Neonatal hypoxic-ischemic encephalopathy (HIE) is a severe neurological disorder caused by impaired cerebral oxygenation and perfusion during the perinatal period (1, 2). It remains a major cause of neonatal death and long-term neurodevelopmental impairment worldwide (3, 4). Affected neonates have increased risks of cerebral palsy, epilepsy, cognitive dysfunction, motor disability, and behavioral problems (4, 5). Advances in perinatal care, neonatal intensive care, and neuroprotection have improved outcomes, but early identification of high-risk neonates remains difficult. Current assessment relies on perinatal history, neurological examination, Apgar score, electroencephalography, neuroimaging, and biochemical indicators (4, 6). These approaches can be constrained by delayed availability, specialized equipment, interobserver variability, or limited sensitivity during the early injury phase. Accessible blood biomarkers may therefore improve early diagnosis and risk stratification in HIE.

Peripheral blood biomarkers are attractive for early detection and serial monitoring of neonatal hypoxic-ischemic brain injury (6–10). Blood sampling is relatively accessible and permits repeated assessment during the evolving phase of neonatal encephalopathy (6, 9). Previous studies have evaluated circulating neuronal injury proteins, inflammatory mediators, and other blood-based biomarkers in neonates with HIE, with several markers showing associations with brain injury severity or clinical outcomes (7–10). Nevertheless, no single blood biomarker has demonstrated sufficient robustness for routine stand-alone clinical use (9). Because HIE involves hypoxia-ischemia, inflammation, vascular dysfunction, blood-brain barrier disruption, coagulation abnormalities, and secondary neuronal injury, markers reflecting these processes may aid disease identification and severity assessment (2, 4, 10, 11).

High-throughput transcriptomic analysis can identify disease-associated molecular signatures and biomarker candidates. Importantly, transcriptomic profiling has previously been applied directly to whole blood from neonates with HIE, demonstrating disease-associated expression changes and supporting the feasibility of blood-based transcriptomic discovery in this setting (12). Public gene-expression datasets are useful for discovery, but cross-dataset analysis must respect biological boundaries. In this study, GSE121178 (12, 13) was derived from human whole blood, whereas GSE18356 (14) and GSE37777 (15) were derived from rat cerebral cortex. Species and tissue differences were treated as biological variation, not as removable batch effects (16). The revised analytical framework therefore did not merge these matrices or use ComBat to claim cross-species comparability. Each dataset was analyzed separately, and rat signals were considered only after one-to-one human ortholog mapping.

After dataset-specific differential analysis, candidate genes were prioritized with a conservative rule set. Human blood evidence was treated as primary when platform annotation allowed direct assessment. Rat cortex results were used only as supportive, directionally concordant biological context. Clinical relevance required validation in an independent human PBMC cohort. This framework acknowledges the scarcity of neonatal HIE human blood transcriptomic datasets without assuming that rat cortex and human blood are equivalent transcriptomic materials.

F3 encodes tissue factor, an integral membrane protein and a principal initiator of the extrinsic coagulation pathway (17). Beyond hemostasis, tissue factor links coagulation with inflammatory and endothelial signaling and can contribute to thrombo-inflammatory vascular responses (18). These mechanisms are relevant to HIE, in which hypoxic-ischemic injury is accompanied by inflammatory activation, vascular and blood-brain barrier dysfunction, coagulation disturbances, and secondary neuronal injury (4, 10, 11, 19). Increased F3 expression may therefore reflect coagulation-inflammation activation during neonatal hypoxic-ischemic brain injury.

Bioinformatics can nominate biomarker candidates, but clinical validation is needed before translation. We therefore measured F3 expression in PBMCs from an independent cohort of neonates with HIE and controls. F3 mRNA was assessed by qRT-PCR, and flTF protein was evaluated by immunofluorescence and Western blotting. We also examined diagnostic performance by ROC analysis and explored the association between F3/flTF expression and disease severity.

Public human neonatal HIE blood transcriptomic datasets are limited in both number and sample size. Experimental hypoxic-ischemic injury datasets were therefore analyzed separately to provide supportive biological context. These datasets were not treated as substitutes for human peripheral blood data. Instead, they were used to determine whether candidate genes showed directionally concordant changes in hypoxic-ischemic injury-related settings.

This study aimed to identify and validate a peripheral blood biomarker candidate for HIE by combining dataset-specific bioinformatics with clinical validation. We tested whether F3 was differentially expressed in HIE, assessed its diagnostic performance, and examined its association with clinical severity. The results may clarify whether F3/flTF deserves further evaluation as a minimally invasive marker for early HIE identification and risk stratification.

## Materials and methods

### Study design

This study used dataset-specific bioinformatics analysis followed by clinical validation to identify blood-based biomarker candidates associated with HIE.

Because public human neonatal HIE blood transcriptomic datasets are scarce, two experimental HIE datasets were included as supportive resources. Given the differences in species and tissue source, these datasets were analyzed independently and were not pooled with the human whole-blood dataset. Rat genes were mapped to one-to-one human orthologs only after dataset-specific differential expression analysis. Rat cortex datasets provided orthology-mapped biological background and were not used to replace human blood validation or to claim direct comparability with human whole blood. Candidate prioritization was followed by validation using PBMCs from neonates with HIE and controls. Diagnostic value was evaluated by ROC curve analysis, and associations with clinical severity indicators were explored.

### Acquisition of public transcriptomic datasets

Gene-expression datasets related to HIE were downloaded from the GEO database. Three independent datasets were included: GSE121178 (12, 13), GSE18356 (14), and GSE37777 (15). GSE121178 is a human whole-blood ceRNA microarray dataset with 3 HIE and 3 non-HIE neonatal samples. GSE18356 and GSE37777 are rat cerebral cortex microarray datasets generated after neonatal hypoxic-ischemic injury. GSE18356 includes control + saline, control + MCI-186, Rice + saline, and Rice + MCI-186 strata. The primary model comparison was Rice + saline versus control + saline, and all Rice versus all control samples were used only as a sensitivity comparison. GSE37777 includes 4 Rice-model and 4 control rat cortex samples.

Expression matrices and sample annotations were obtained for each dataset. Samples were assigned to control or HIE/hypoxic-ischemic injury groups according to the original dataset information. Probes were annotated to official gene symbols using the corresponding platform files. When several probes mapped to the same gene symbol, their average expression value was used for downstream analysis.

### Dataset-specific preprocessing and normalization

Raw or processed gene-expression matrices were imported into R for preprocessing. Expression values were log2-transformed when needed, and platform-recommended normalized matrices were used when available. Probe annotation followed the corresponding GEO platform files. Because GSE121178, GSE18356, and GSE37777 differ by species and tissue source, their expression matrices were not merged before differential expression analysis.

Quality assessment was performed separately for each dataset, including inspection of expression distributions and sample-level structure when appropriate. Cross-species and cross-tissue differences were treated as biological context rather than technical batch effects (16). ComBat-based correction across human whole blood and rat cerebral cortex datasets was not used in the revised primary analysis. Pooled PCA or pooled boxplots were also not used to support biological conclusions.

### Identification of differentially expressed genes

Differential expression analysis was performed within each dataset using limma or another linear-model framework suited to the platform and comparison structure. Benjamini-Hochberg FDR correction was applied separately within each dataset. Because the available public HIE datasets were small, the cross-dataset screen used an exploratory nominal threshold of |log2FC| >= 1 and nominal *P* < 0.05 to define differentially expressed candidate genes. These genes were not interpreted as transcriptome-wide FDR-significant DEGs unless the adjusted *P* value also met the FDR threshold. For F3, both nominal and adjusted *P* values were reported. The human discovery signal was treated as exploratory because F3 did not reach FDR significance in GSE121178.

Dataset-specific volcano plots, candidate-gene-count summaries, UpSet analysis, log2FC consistency heatmaps, and F3 expression plots were used to present within-dataset results. Merged heatmaps, merged PCA plots, and a single unified gene count across human blood and rat cortex were not used as primary evidence. Complete candidate-gene results, probe-to-gene annotations, and ortholog mapping information are provided in the supplementary materials when available.

### Ortholog mapping and candidate prioritization

After dataset-specific differential analysis, rat genes were mapped to one-to-one human orthologs before comparison with human gene symbols. Probe-to-gene and ortholog mapping information was retained for supplementary reporting. Candidate prioritization followed a prespecified hierarchy: (1) nominal differential expression in the human whole-blood dataset, with adjusted *P* values reported for transparency; (2) directionally concordant rat cortex evidence after ortholog mapping; (3) overlap with HIE-associated disease-gene resources; and (4) validation in independent human PBMC samples. F3-specific estimates, including log2FC, nominal *P* value, adjusted *P* value, direction, and sample size, were reported for all three datasets.

### Collection of HIE-related genes from public databases

Disease-associated candidate genes were collected by searching GeneCards, Online Mendelian Inheritance in Man (OMIM), and the Therapeutic Target Database (TTD) using the keywords “neonatal hypoxic-ischemic encephalopathy,” “HIE,” and related terms. Genes retrieved from the three databases were merged, and duplicates were removed.

Genes obtained from GeneCards, OMIM, and TTD were compared using a Venn diagram. Disease-related genes were then compared with dataset-specific human genes and rat-to-human orthologs generated after differential analysis. A gene was prioritized only after species/tissue context, effect direction, FDR, and validation status were considered.

### Prioritization of F3 as the primary candidate gene

F3 was retained as an exploratory candidate because it showed nominal upregulation in the human whole-blood dataset, directionally concordant upregulation in both rat cortex datasets after ortholog mapping, overlap with HIE-related disease-gene resources, and marked F3/flTF upregulation in the independent human PBMC validation cohort. This prioritization does not imply direct comparability between rat cortex and human blood expression matrices. It also does not establish F3 as an independent diagnostic biomarker.

### Clinical sample collection

Peripheral blood samples were collected from neonates diagnosed with HIE and from control neonates without evidence of cerebral ischemic injury at the Affiliated Hospital of Putian University, China. HIE was diagnosed using clinical manifestations, perinatal history, neurological examination, and auxiliary examinations, including neuroimaging and electroencephalographic (EEG) findings when available. Sample and clinical data collection was conducted under an approved neonatal HIE research project supported by the Putian Medical and Health Science and Technology Innovation Joint Fund (Grant No. 2024SJYL072). The study was approved by the Ethics Committee of the Affiliated Hospital of Putian University (Approval No.: 202599). Written informed consent was obtained from the legal guardians of all enrolled neonates before sample collection.

Non-HIE control neonates were selected from infants without hypoxic-ischemic brain injury, severe infection, congenital neurological disease, chromosomal abnormality, or major organ dysfunction. Clinical information was collected from medical records, including gestational age, birth weight, sex, Apgar score, laboratory indicators, neuroimaging findings, treatment information, sampling time after birth, and disease severity classification (Table 1).

**Table 1:** Baseline clinical characteristics of HIE and controls.

Variables	HIE group (*n* = 40)	Control group (*n* = 30)	*P* value
Male sex, *n* (%)	23 (57.5)	16 (53.3)	0.73
Gestational age, weeks	38.4 ± 1.7	38.7 ± 1.5	0.45
Birth weight, g	3120 ± 520	3235 ± 485	0.35
Cesarean delivery, *n* (%)	18 (45.0)	13 (43.3)	0.89
1-min Apgar score	4.0 (3.0–6.0)	9.0 (8.0–10.0)	<0.001
5-min Apgar score	6.0 (5.0–8.0)	10.0 (9.0–10.0)	<0.001
Sampling time after birth, h	18.0 (10.0–30.0)	20.0 (12.0–32.0)	0.58
HIE grade, *n* (%)			—
Mild	12 (30.0)	—	—
Moderate	18 (45.0)	—	—
Severe	10 (25.0)	—	—
Seizure, *n* (%)	17 (42.5)	0 (0.0)	<0.001
Mechanical ventilation, *n* (%)	14 (35.0)	0 (0.0)	<0.001
Therapeutic hypothermia, *n* (%)	16 (40.0)	0 (0.0)	<0.001
confirmed infection, *n* (%)	6 (15.0)	2 (6.7)	0.29
Lactate, mmol/L	5.8 (4.2–8.1)	1.9 (1.4–2.5)	<0.001
Arterial pH	7.12 ± 0.10	7.35 ± 0.05	<0.001
Base deficit, mmol/L	12.6 ± 4.8	3.2 ± 1.7	<0.001
WBC, ×10^9/L	16.8 ± 5.6	11.5 ± 3.2	<0.001
CRP, mg/L	7.8 (3.2–15.6)	2.1 (1.0–4.3)	0.002
PCT, ng/mL	0.68 (0.28–1.45)	0.16 (0.08–0.35)	<0.001
PT, s	15.8 ± 2.7	13.6 ± 1.5	<0.001
APTT, s	46.2 ± 9.8	38.5 ± 6.4	<0.001
INR	1.28 ± 0.22	1.08 ± 0.13	<0.001
Fibrinogen, g/L	2.1 ± 0.7	2.6 ± 0.6	0.003
D-dimer, mg/L FEU	1.85 (0.96–3.40)	0.42 (0.25–0.78)	<0.001

Continuous variables are presented as mean ± SD or median (interquartile range), depending on data distribution. Categorical variables are presented as *n* (%). WBC, white blood cell count; CRP, C-reactive protein; PCT, procalcitonin; PT, prothrombin time; APTT, activated partial thromboplastin time; INR, international normalized ratio; FEU, fibrinogen equivalent units.

#### Inclusion and exclusion criteria

The inclusion criteria for the disease group were as follows:

1. neonates diagnosed with mild, moderate, or severe HIE according to the Sarnat and Sarnat staging system (20), based on evidence of perinatal asphyxia and abnormal neurological examination findings;
2. availability of peripheral blood samples;
3. complete basic clinical information;
4. no prior treatment likely to substantially affect gene expression before sample collection; blood sampling was performed within 6 h of enrollment and before therapeutic hypothermia when applicable.

The inclusion criteria for the control group were as follows:

1. neonates without clinical or imaging evidence of cerebral ischemic injury;
2. availability of peripheral blood samples;
3. no severe systemic disease or congenital abnormality.

The exclusion criteria were as follows:

1. congenital malformations or chromosomal abnormalities;
2. inherited metabolic disease;
3. severe systemic infection or sepsis at the time of sampling;
4. severe hepatic, renal, or cardiac dysfunction;
5. incomplete clinical data;
6. inadequate blood sample quality.

### RNA extraction and reverse transcription

Total RNA was extracted from freshly isolated PBMCs using TRIzol reagent (Invitrogen, Thermo Fisher Scientific, Waltham, MA, USA) according to the manufacturer's instructions. RNA concentration and purity were assessed spectrophotometrically (A260/A280 ratio ≥ 1.8). Complementary DNA (cDNA) was synthesized from 1 *μ*g of total RNA using PrimeScript™ RT Reagent Kit with gDNA Eraser (Takara Bio, Kusatsu, Japan) according to the manufacturer's protocol. Synthesized cDNA was stored at −20 °C until use.

### Quantitative real-time PCR (qRT-PCR)

qRT-PCR was performed using TB Green™ Premix Ex Taq™ II (Takara Bio, Kusatsu, Japan) on a QuantStudio™ 7 Flex Real-Time PCR System (Applied Biosystems, Thermo Fisher Scientific, Waltham, MA, USA). The thermal cycling conditions were: initial denaturation at 95 °C for 10 min, followed by 40 cycles of 95 °C for 15 s and 60 °C for 60 s. Primer sequences used for amplification of F3 (flTF mRNA) and GAPDH (internal reference) are listed in Table 2. Each reaction was performed in triplicate. Relative mRNA expression was calculated using the 2^−*ΔΔ*Ct^ method, normalized to GAPDH, and expressed as fold change relative to the control group mean.

**Table 2:** Primer sequences used for qRT-PCR.

Gene	Primer	Sequence (5'→3’)	Amplicon (bp)
F3 (flTF)	Forward	5'-GCCAGGAGAAAGGGGAATCA-3’	150 bp
	Reverse	5'-CAGTGCAATATAGCATTTGCAGTAGC-3'	150 bp
GAPDH	Forward	5'-GGAGCGAGATCCCTCCAAAAT-3'	197 bp
	Reverse	5'-GGCTGTTGTCATACTTCTCATGG-3'	197 bp

### Diagnostic performance of PBMC F3 mRNA

ROC curve analysis was performed to evaluate the diagnostic value of PBMC F3 mRNA expression for identifying HIE. Relative F3 expression measured by qRT-PCR and normalized to the internal control was used as the input variable. Diagnostic group (HIE vs. non-HIE control) was used as the binary outcome.

The ROC curve was generated by plotting sensitivity against 1-specificity across possible threshold values. The AUC quantified the discriminatory performance of F3, with 1.0 indicating perfect discrimination and 0.5 indicating no diagnostic utility.

The optimal cutoff for PBMC F3 mRNA expression was determined using the Youden index (J = sensitivity + specificity - 1). This index identifies the threshold that maximizes the sum of sensitivity and specificity. Analyses and visualization were performed in R software (version 4.4.3) using the pROC and ggplot2 packages. A two-sided *P* < 0.05 was considered statistically significant.

### Blood collection and PBMC isolation

Peripheral venous blood (2–3 mL) was collected from each enrolled neonate (HIE group, *n* = 40; non-HIE control group, *n* = 30) into K2EDTA anticoagulant tubes (BD Biosciences, Franklin Lakes, NJ, USA) within 6 h after study enrollment. Because enrollment occurred after the initial clinical evaluation for suspected HIE, sampling time was also recorded as hours after birth. The median sampling time after birth was 18.0 h in the HIE group and 20.0 h in the non-HIE control group (Table 1). Blood sampling was performed before therapeutic hypothermia when applicable, as specified in the inclusion criteria.

### Immunofluorescence staining

PBMCs (5 × 10^4/coverslip) were seeded onto poly-L-lysine-coated coverslips (Sigma-Aldrich) and allowed to adhere for 1 h at 37 °C. Cells were fixed with 4% paraformaldehyde (Sigma-Aldrich) for 15 min at room temperature without permeabilization to preserve membrane integrity for surface flTF detection. Non-specific binding was blocked with 5% bovine serum albumin (BSA; Sigma-Aldrich) in PBS for 30 min. Coverslips were incubated overnight at 4 °C with rabbit anti-human tissue factor/CD142 monoclonal antibody (clone EPR22548-232, ab228968, 1:200; Abcam, Cambridge, UK). After washing, coverslips were incubated with Alexa Fluor 488-conjugated goat anti-rabbit IgG secondary antibody (1:500; Invitrogen, Thermo Fisher Scientific) for 45 min in the dark, then mounted with ProLong Gold Antifade Mountant with DAPI (Invitrogen). Images were acquired by confocal laser scanning microscopy. When multiple microscopic fields were acquired from the same participant, the average MFI per participant was used as the statistical unit to reduce pseudo-replication.

### Western blot analysis

PBMCs were lysed in RIPA buffer (Beyotime Biotechnology, Shanghai, China) supplemented with protease inhibitor cocktail (cOmplete Mini; Roche Diagnostics, Basel, Switzerland) on ice for 30 min. Total protein was quantified by BCA assay (Beyotime Biotechnology). Equal amounts of protein were resolved by 10% SDS-PAGE and transferred onto PVDF membranes. Membranes were blocked with 5% non-fat milk/TBST for 1 h, then incubated overnight at 4 °C with anti-tissue factor/CD142 (clone EPR22548-240, ab252918，1:1,000; Abcam; flTF expected at ∼47 kDa) and anti-GAPDH (clone 1E6D9, 1:5,000; Proteintech Group). Signals were developed using enhanced chemiluminescence and quantified by densitometry. Western blot data were analyzed at the biological-sample level. Biological sample numbers and lane-level source data are provided in the supplementary source-data file.

### Correlation between F3 expression and clinical severity

HIE severity was classified as mild, moderate, or severe according to local institutional criteria for neonatal encephalopathy. Before analysis, these criteria were reviewed and confirmed to align with major domains of Sarnat-based assessment (20), including consciousness, spontaneous activity, muscle tone, primitive reflexes, autonomic function, seizures, respiratory support, and available EEG/aEEG or neuroimaging findings (Supplementary Table S3).

Severity grading was completed within the first 24 h after birth by the clinical research team. Two experienced neonatologists independently reviewed the clinical records and bedside neurological assessments and assigned the HIE severity grade. Discrepant grades were resolved by discussion and clinical adjudication. Severity assessment was completed before biomarker-based statistical analysis, and clinicians involved in severity classification did not perform the laboratory measurement of F3/flTF expression.

For continuous variables, Pearson or Spearman correlation analysis was performed depending on data distribution. For categorical severity groups, F3 expression levels were compared using Student's t-test, Mann–Whitney U test, one-way analysis of variance, or Kruskal–Wallis test, as appropriate. Potential confounding factors, including gestational age, sex, sampling time after birth, WBC, CRP, PCT, PT, APTT, INR, fibrinogen, D-dimer, seizure, mechanical ventilation, therapeutic hypothermia, and confirmed infection, were considered clinically relevant. The present cohort was not sufficiently powered to determine whether F3 is independently associated with HIE after adjustment for inflammatory and coagulation-related confounders.

### Statistical analysis

All statistical analyses were performed using R software and/or SPSS software. Continuous variables were presented as mean ± standard deviation for normally distributed data or median with interquartile range for non-normally distributed data. Categorical variables were expressed as frequencies and percentages.

Comparisons between two groups were performed using Student's t-test or Mann–Whitney U test for continuous variables and chi-square test or Fisher's exact test for categorical variables. For comparisons among multiple groups, one-way analysis of variance followed by Tukey's *post-hoc* test or Kruskal–Wallis test was used, as appropriate. Differential expression analysis was corrected for multiple testing using the Benjamini-Hochberg method within each dataset. A two-sided *P*-value < 0.05 was considered statistically significant for clinical analyses.

## Results

### Redefined cross-dataset analysis strategy

**Figure 1:**
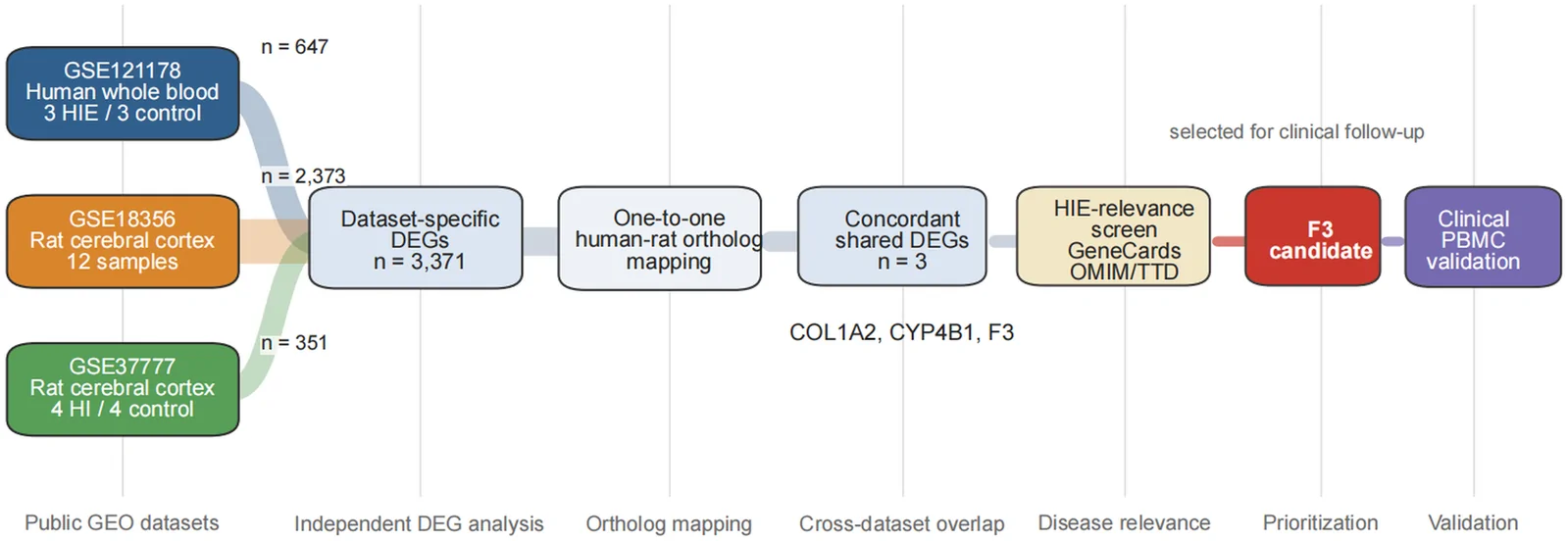
Sankey diagram illustrating the stepwise screening process for candidate HIE-related genes. Public GEO datasets were analyzed independently. Rat cortex data were used as supportive cross-species evidence only after dataset-specific differential testing and one-to-one human-rat ortholog mapping. Nominal screening threshold: *P* value < 0.05 and |log2FC| >= 1.

The three public GEO datasets differed by species and tissue source, so they were analyzed as separate discovery resources rather than as batches of a single expression matrix. GSE121178 was treated as a human whole-blood dataset. GSE18356 and GSE37777 were treated as rat cerebral cortex datasets. The revised workflow used dataset-specific preprocessing and differential analysis, followed by rat-to-human one-to-one ortholog mapping, overlap analysis, HIE-related disease-gene screening, and clinical PBMC validation (Figure 1).

### Independent candidate-gene landscapes and cross-dataset candidate overlap

**Figure 2:**
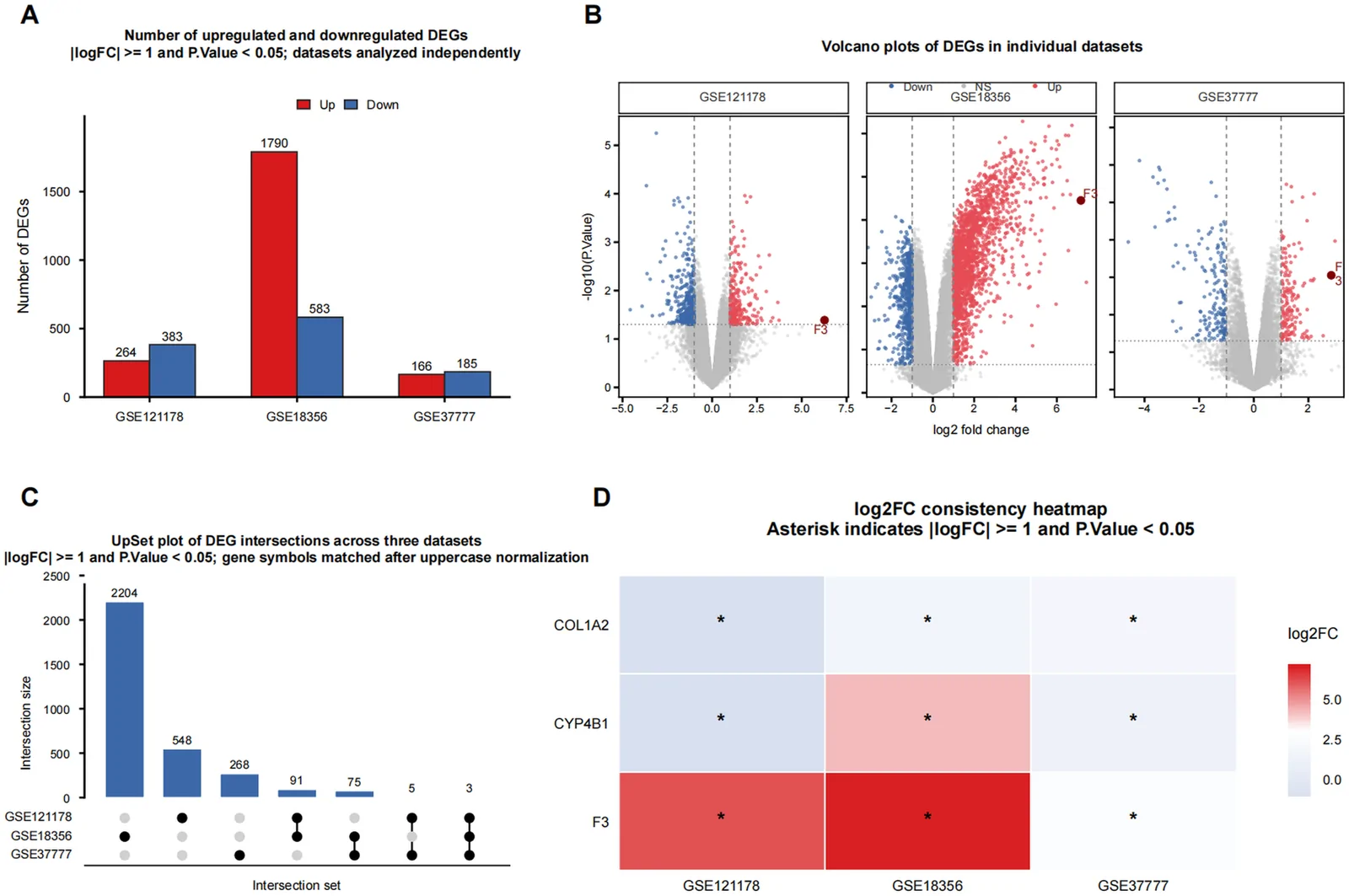
Independent candidate-gene landscapes and cross-dataset candidate overlap. **(A)** Number of upregulated and downregulated nominally differentially expressed candidate genes in each dataset. **(B)** Dataset-specific volcano plots; F3 is highlighted in each panel. **(C)** UpSet plot showing candidate-gene intersections after gene-symbol harmonization and post-analysis ortholog mapping. **(D)** log2FC consistency heatmap for COL1A2, CYP4B1, and F3. Asterisks indicate genes meeting |log2FC| >= 1 and nominal *P* value < 0.05 in the indicated dataset.

Using the prespecified exploratory nominal threshold of |log2FC| >= 1 and nominal *P* < 0.05, dataset-specific analysis identified 647 candidate genes in GSE121178, 2,373 in GSE18356, and 351 in GSE37777. These sets are described as nominally differentially expressed candidate genes, not as strictly FDR-significant DEGs. Upregulated and downregulated candidate genes numbered 264 and 383 in GSE121178, 1,790 and 583 in GSE18356, and 166 and 185 in GSE37777, respectively (Figure 2A). Dataset-specific volcano plots showed the distribution of differential signals in each dataset and highlighted F3 without merging expression matrices across species or tissues (Figure 2B).

After rat-to-human ortholog mapping and gene-symbol harmonization, overlap analysis identified three concordant genes shared across all three nominal candidate sets: COL1A2, CYP4B1, and F3 (Figure 2C). A log2FC consistency heatmap showed a positive F3 signal in all three datasets (Figure 2D). Because GSE121178 included only 3 HIE and 3 control samples, and because F3 did not reach FDR significance in that dataset, this evidence was interpreted as exploratory prioritization rather than definitive transcriptomic discovery.

Among the three concordant overlapping candidates, F3 was selected for clinical validation using a predefined biological and translational rationale. F3 showed directionally concordant upregulation across the human whole-blood dataset and the two supportive rat cortex datasets. It encodes tissue factor, an initiator of the extrinsic coagulation pathway and a mediator of coagulation-inflammatory crosstalk, endothelial activation, microvascular dysfunction, and ischemia-related tissue injury (17, 18). These processes are relevant to neonatal HIE (4, 10, 11, 19). F3 was also present in HIE-related disease-gene resources. Because tissue factor can be measured at both mRNA and protein levels in peripheral blood-derived cells, F3 was considered suitable for PBMC-based validation. COL1A2 and CYP4B1 were retained as overlapping candidates but were not selected for experimental validation in this study because their links to acute coagulation-inflammatory activation and peripheral blood-based HIE risk stratification were less direct.

### Dataset-specific F3 expression supports concordant upregulation

**Figure 3:**
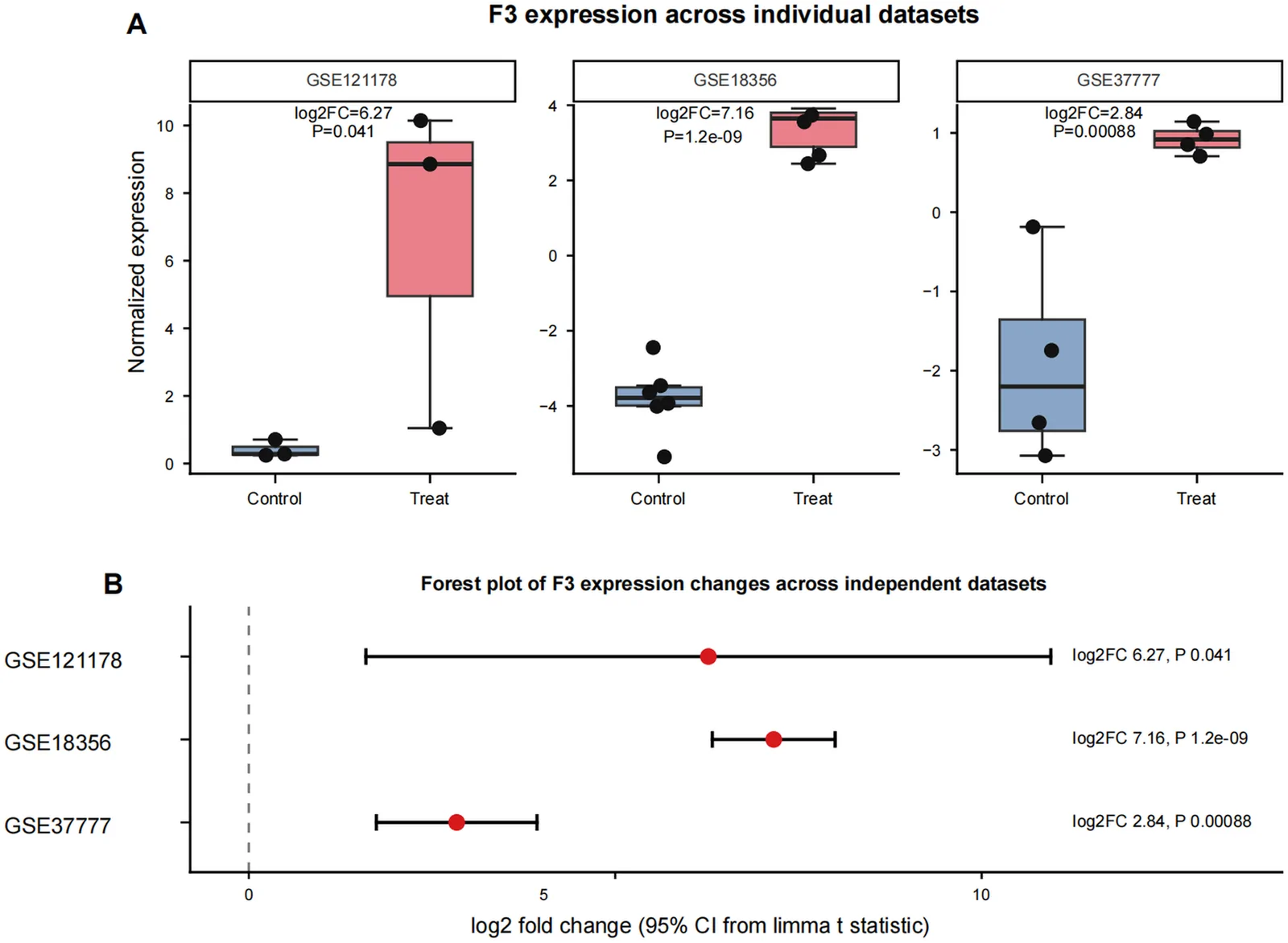
Dataset-specific F3 expression supports concordant upregulation. **(A)** Box-and-point plots showing normalized F3 expression in GSE121178, GSE18356, and GSE37777 analyzed separately. Dataset labels show log2FC and nominal *P* values. **(B)** Forest plot summarizing F3 log2 fold changes and approximate 95% confidence intervals across independent datasets. Rat cortex datasets are interpreted as supportive cross-species evidence, not as direct substitutes for human blood validation.

F3 expression was evaluated separately in each dataset to avoid obscuring dataset-specific patterns across species or tissues. In GSE121178 human whole blood, F3 showed nominal upregulation in HIE compared with non-HIE controls (log2FC = 6.27, nominal *P* = 0.041, adjusted *P* = 0.524; *n* = 3/3), but it did not reach FDR significance. In GSE18356 rat cortex, F3 was strongly upregulated after hypoxic-ischemic injury (log2FC = 7.16, nominal *P* = 1.25e−09, adjusted *P* = 9.43e−08; *n* = 6/6). In GSE37777 rat cortex, F3 was also upregulated (log2FC = 2.84, nominal *P* = 8.85e-04, adjusted *P* = 0.069; *n* = 4/4). These findings support exploratory prioritization of F3 while preserving the distinction between human blood and rat cortex (Figure 3).

### Functional enrichment analysis

**Figure 4:**
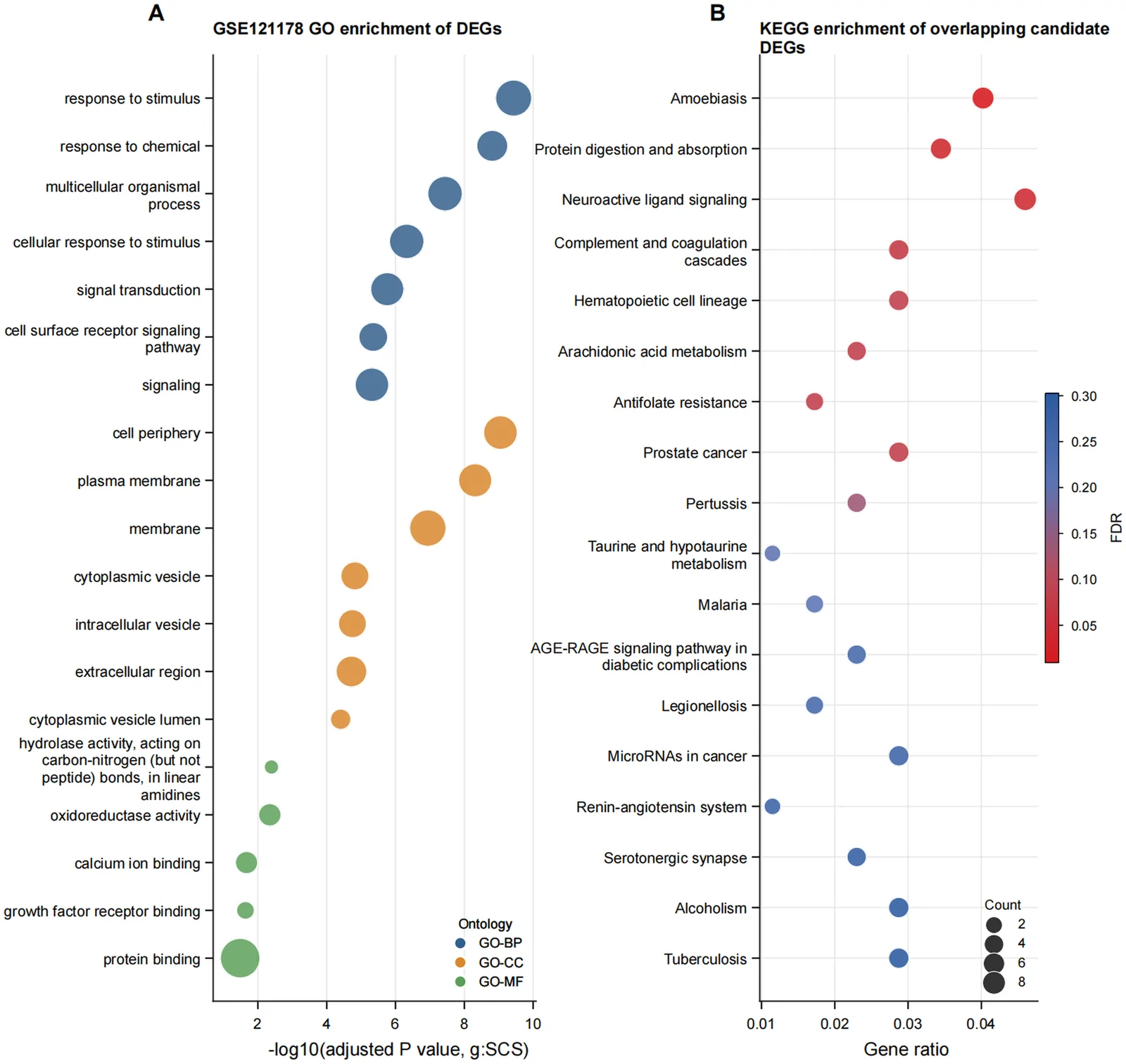
Exploratory functional context for HIE-related candidate genes. **(A)** GO analysis of GSE121178 human whole-blood candidate genes. Color indicates ontology category and bubble size denotes gene count. **(B)** Candidate pathway annotation using genes shared by at least two datasets. Color indicates FDR and bubble size denotes gene count. These pathway results are intended as biological context and should not be interpreted as confirmatory enrichment based on the three-gene core overlap alone.

Exploratory pathway analysis was performed to provide biological context. The input gene sets were GSE121178 human whole-blood candidate genes and the broader set of candidate genes shared by at least two datasets. GO analysis of GSE121178 candidate genes identified terms related to response to stimulus, response to chemical, signal transduction, membrane or cell periphery, vesicle-related cellular components, protein binding, calcium ion binding, and oxidoreductase activity (Figure 4A). The KEGG panel should be interpreted as candidate pathway annotation rather than definitive pathway enrichment, because the cross-dataset overlap was small and sensitive to the input gene set (Figure 4B). Direction-specific GO enrichment for GSE121178 is provided in Supplementary Figure S1.

Direction-specific GO enrichment in GSE121178 is shown in Supplementary Figure S1. Top GO-BP, GO-CC, and GO-MF terms are presented separately for upregulated and downregulated DEGs.

### Clinical validation of F3 mRNA expression and diagnostic performance

**Figure 5:**
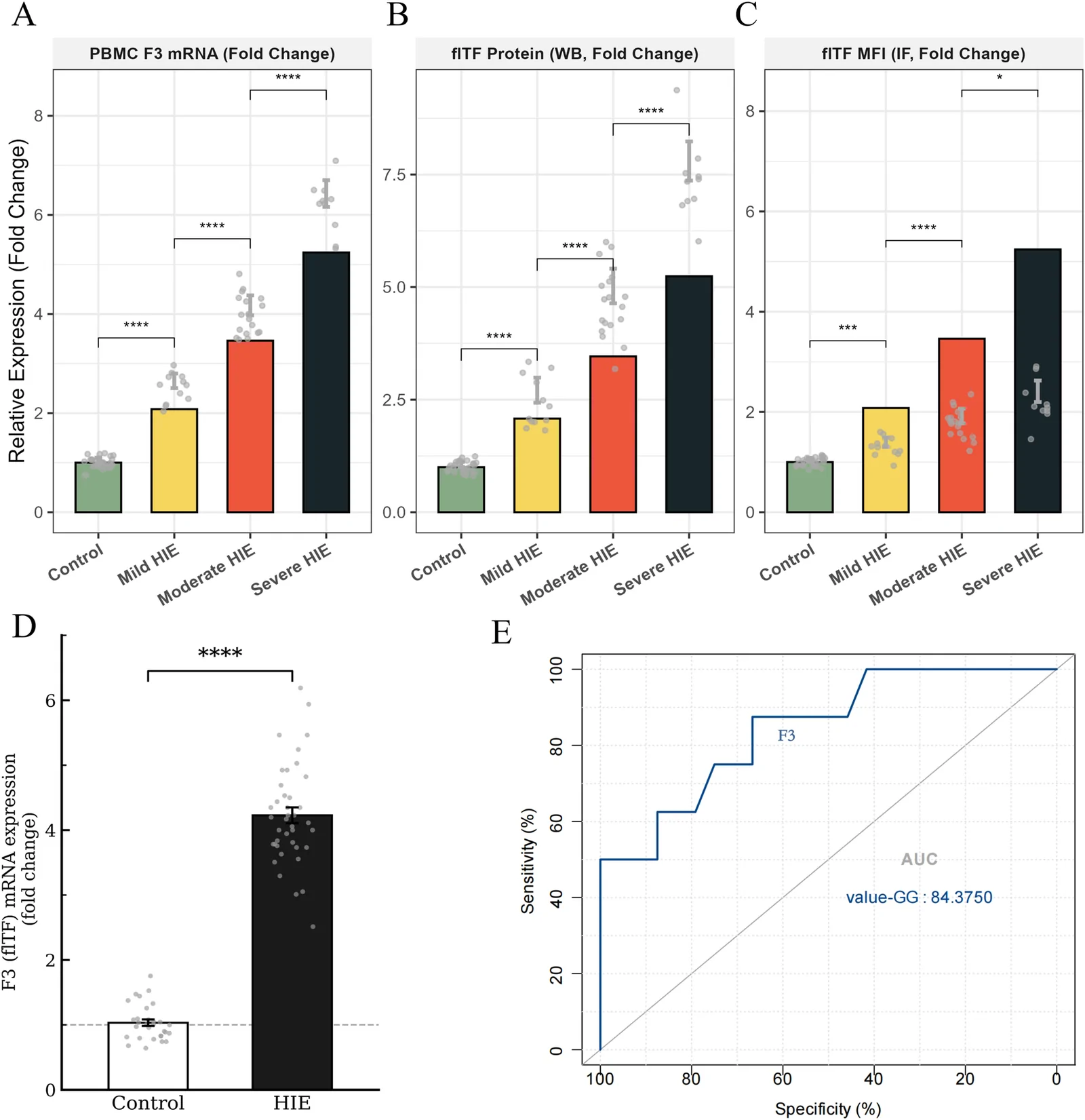
PBMC F3/flTF expression increases with HIE severity and discriminates HIE from controls. **(A)** PBMC F3 mRNA expression across control, mild HIE, moderate HIE, and severe HIE groups. **(B)** flTF protein abundance measured by Western blot across severity groups. **(C)** flTF mean fluorescence intensity measured by immunofluorescence across severity groups. **(D)** PBMC F3 mRNA expression in all HIE neonates compared with controls. **(E)** ROC curve for PBMC F3 mRNA in discriminating HIE from controls. AUC = 0.844 (95% CI: 0.735−0.953). Data are shown with individual sample points; *P < 0.05, ***P < 0.001, ****P < 0.0001.

Peripheral blood samples were collected from neonates with HIE and from non-HIE control neonates to validate the candidate identified by dataset-specific transcriptomic prioritization and disease-gene context. qRT-PCR confirmed higher PBMC F3 mRNA expression in neonates with HIE than in non-HIE controls (4.2 +/- 0.2 vs. 1.0 +/- 0.1; *P* < 0.0001; Figure 5D). ROC analysis showed good discrimination in this single-center cohort, with an AUC of 0.844 (95% CI, 0.735–0.953), sensitivity of 75.0%, and specificity of 80.0% at the optimal Youden-index cutoff (Figure 5E). This result supports F3/flTF as a clinically evaluated candidate marker that requires external validation, not as a stand-alone diagnostic test.

### PBMC F3/flTF expression is positively associated with HIE clinical severity

HIE neonates were stratified by clinical severity as mild (*n* = 12, 30.0%), moderate (*n* = 18, 45.0%), or severe (*n* = 10, 25.0%). PBMC F3 mRNA expression increased from mild to severe HIE (2.5 +/- 0.4, 4.1 +/- 0.5, and 6.4 +/- 0.6, respectively; F = 148.5, *P* < 0.0001). Similar protein-level gradients were observed for flTF abundance measured by Western blot (2.6 +/- 0.5, 4.4 +/- 0.7, and 7.0 +/- 0.9-fold vs. controls; F = 112.3, *P* < 0.0001) and for flTF mean fluorescence intensity by immunofluorescence (F = 45.8, *P* < 0.0001; Figures 5A–C; Table 3).

**Table 3:** Association of PBMC F3 mRNA and protein expression with HIE clinical severity.

Clinical Group	Sample (n, %)	PBMC F3 mRNA (Fold Change)	flTF Protein (WB, Fold Change)	flTF MFI (IF, fold change)
Control	30 (100.0%)	1.0 ± 0.1	1.0 ± 0.1	1.0 ± 0.1
Mild HIE	12 (30.0%)	2.5 ± 0.4	2.6 ± 0.5	1.3 ± 0.2
Moderate HIE	18 (45.0%)	4.1 ± 0.5	4.4 ± 0.7	1.7 ± 0.3
Severe HIE	10 (25.0%)	6.4 ± 0.6	7.0 ± 0.9	2.2 ± 0.4
Statistic (F/H)	-	F = 148.5	F = 112.3	F = 45.8
*P*-value	-	<0.0001	<0.0001	<0.0001

### Protein-level validation of flTF upregulation in PBMCs

**Figure 6:**
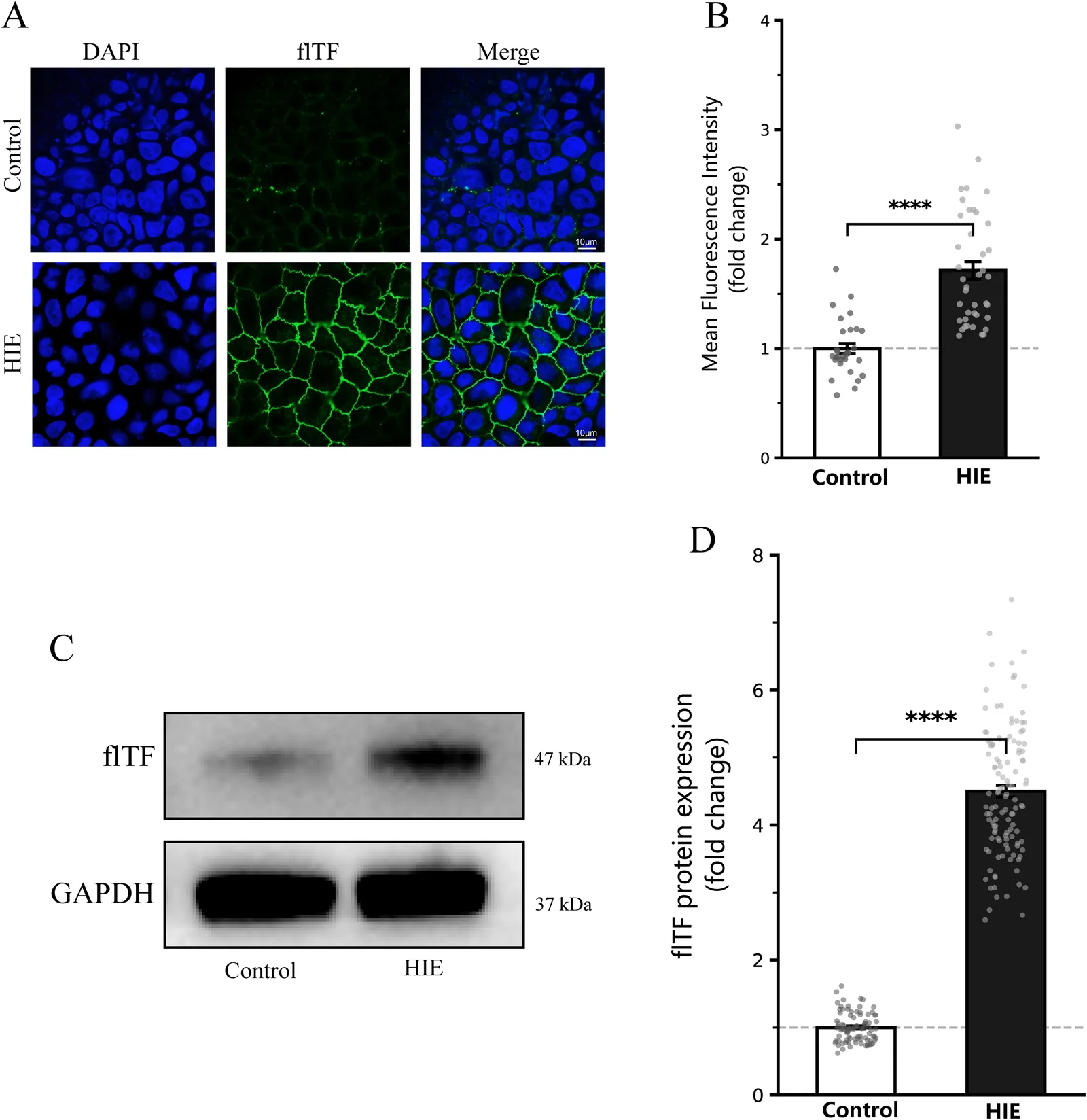
flTF protein is upregulated in PBMCs from HIE (Control, *n* = 30; HIE, *n* = 40). **(A)** Representative confocal images (DAPI, flTF, merge); HIE shows prominent ring-pattern membrane flTF staining. Scale bar, 10 μm. **(B)** flTF MFI quantification (fold change vs. control mean). **(C)** Representative Western blot of flTF (47 kDa) and GAPDH (37 kDa, loading control) in Control and HIE PBMCs. **(D)** Densitometric quantification of flTF/GAPDH (fold change). **(B,D)** Mea*n* ± SEM; dashed line, baseline (fold change = 1.0); ****P < 0.0001, Mann–Whitney U test.

Immunofluorescence staining showed stronger flTF expression in the HIE group than in non-HIE controls, with membrane-associated signal along the plasma membrane (Figure 6A). Quantitative analysis confirmed increased flTF mean fluorescence intensity in the HIE group (approximately 1.7-fold vs. control; *P* < 0.0001, Mann–Whitney U test; Figure 6B). Western blotting detected flTF as an approximately 47-kDa band consistent with tissue factor (Figure 6C). Densitometry normalized to GAPDH (37 kDa) showed higher flTF protein in HIE samples (*P* < 0.0001; Figure 6D). These protein data support the mRNA findings. Biological sample numbers, lane-level structure, and field-to-sample aggregation are provided in the source-data file.

## Discussion

This study used dataset-specific bioinformatics and clinical validation to evaluate F3/flTF as a peripheral blood candidate marker for HIE. Because the public datasets differed by species and tissue source, human whole-blood and rat cerebral cortex expression matrices were not directly pooled. Each public dataset was interpreted within its own biological context, rat genes were considered only after ortholog mapping, and the central clinical evidence came from an independent human PBMC cohort. This analysis prioritized three overlapping candidates, COL1A2, CYP4B1, and F3. F3 was selected for further validation because it showed nominal upregulation in the small human whole-blood dataset, directionally concordant rat cortex evidence, disease-gene relevance, and PBMC validation.

A strength of this study is that public transcriptomic evidence was combined with independent clinical validation while preserving cross-species and cross-tissue boundaries. The rat cortex datasets were retained to provide biological background on hypoxic-ischemic injury and F3-related coagulation-inflammatory pathways. They were not used to replace human blood evidence or to claim equivalence between cerebral cortex and peripheral blood transcriptomes. The revised workflow, dataset-specific volcano plots, F3 expression panels, and log2FC consistency heatmap were added to make this distinction explicit.

The prioritization of F3 is biologically plausible. F3 encodes tissue factor, a principal initiator of the extrinsic coagulation pathway (17). Tissue factor also links coagulation with inflammatory and endothelial signaling (18). These processes are relevant to HIE, in which hypoxia-ischemia can trigger inflammatory activation, vascular and blood-brain barrier dysfunction, coagulation abnormalities, microcirculatory impairment, and secondary neuronal damage (4, 10, 11, 19). Elevated F3 expression in PBMCs may therefore reflect coagulation-inflammation activation associated with HIE, whereas rat cortex findings provide supportive mechanistic context only.

COL1A2 and CYP4B1 were also identified as concordant overlapping candidates, but F3 was prioritized because its link to coagulation-inflammatory and vascular injury pathways in neonatal HIE was more direct and because it was feasible for peripheral blood-based validation. COL1A2 is mainly related to extracellular matrix organization and tissue remodeling, whereas CYP4B1 is associated with metabolic and oxidative pathways. These genes may also be relevant to hypoxic-ischemic injury and merit further study. In this exploratory study, however, their relationship with acute PBMC-based risk stratification appeared less direct than that of F3.

Clinical validation provided the strongest evidence for F3 in this study. qRT-PCR showed an approximately 4.2-fold increase in F3 mRNA expression in PBMCs from neonates with HIE. Immunofluorescence and Western blot analyses supported increased flTF protein expression. The membrane-associated staining pattern observed by immunofluorescence is consistent with the localization of full-length tissue factor. These findings indicate that F3 upregulation is detectable in peripheral immune cells from clinically affected neonates, independent of the cross-species transcriptomic prioritization step.

The diagnostic performance of F3 should be interpreted cautiously. ROC analysis showed an AUC of 0.844 for distinguishing neonates with HIE from non-HIE controls in this cohort. F3 should not be considered a stand-alone diagnostic marker at this stage. It may be more useful as part of a combined model that includes clinical history, Apgar score, neurological findings, neuroimaging, electroencephalography, inflammation markers, coagulation indices, and other biochemical indicators.

The association between F3 expression and disease severity is clinically relevant. HIE ranges from mild neurological impairment to severe encephalopathy with seizures, respiratory failure, and long-term neurodevelopmental sequelae (4, 21). Blood-based biomarker studies in neonatal HIE have shown that circulating molecular or biochemical markers can vary with evolving encephalopathy, imaging-defined injury, or clinical severity (7–10, 22). If validated in larger cohorts, F3 may help identify neonates who require closer monitoring or more intensive neuroprotective management.

Several mechanisms may explain increased F3 expression in HIE. Hypoxic-ischemic stress may activate monocytes, endothelial cells, and circulating immune cells, leading to higher tissue factor expression. Tissue factor-mediated thrombin generation can amplify coagulation-inflammatory signaling and vascular dysfunction (17, 18). Coagulation abnormalities are also well recognized in neonates with HIE, particularly in critically ill infants undergoing therapeutic hypothermia (19). Together with the inflammatory and vascular injury pathways implicated in HIE (4, 10, 11), these observations provide biological context for increased PBMC F3 expression. However, the observed increase may partly reflect changes in circulating immune-cell composition or systemic coagulation-inflammatory activation rather than HIE-specific transcriptional regulation. Cell-subset-specific analysis or single-cell validation will be needed to define the cellular source and disease specificity of F3.

This study has several limitations. The public discovery datasets were small and biologically heterogeneous. GSE121178 is human whole blood, whereas GSE18356 and GSE37777 are rat cerebral cortex. Experimental hypoxic-ischemic injury datasets were included because public human neonatal HIE blood transcriptomic data are limited. Species and tissue differences remain major sources of biological heterogeneity. These datasets were therefore used only as supportive, orthology-mapped biological context, and the main clinical relevance of F3/flTF was assessed through independent human PBMC validation.

The transcriptomic screen used a nominal *P*-value threshold to retain candidates in small public datasets, although Benjamini-Hochberg adjusted *P* values were calculated and reported. These results should be interpreted as exploratory. F3 met the nominal threshold in GSE121178 but did not reach FDR significance in that small human dataset. Rat cortex findings are supportive and orthology-mapped, but they cannot establish that peripheral blood and brain tissue share directly comparable expression changes. Pathway analyses are also sensitive to the input gene set and should be viewed as biological context rather than confirmatory pathway evidence.

Other limitations relate to candidate selection and clinical validation. Disease-gene resources may be affected by annotation bias and incomplete coverage, and focusing on F3 may have missed other relevant candidates. The clinical validation cohort was modest and came from a limited clinical population. No prospective power calculation was performed, and larger multicenter human blood cohorts are needed. F3 is closely related to inflammation, coagulation activation, infection, hypoxic stress, seizures, ventilation status, therapeutic hypothermia, and PBMC cell composition. The present cohort was not powered to determine whether F3 is independently associated with HIE after adjustment for these confounders. The sample-level structure of Western blot and immunofluorescence analyses, including biological sample numbers and field-level aggregation, is reported in source-data files when available.

Future studies should prioritize prospective multicenter validation, serial measurement of F3 expression after hypoxic-ischemic insult, and comparison with established clinical and biochemical markers. It will also be important to test whether F3 improves diagnostic accuracy within a multimarker prediction model. Mechanistic studies using cellular and animal models are needed to determine whether F3 directly contributes to endothelial injury, microthrombosis, neuroinflammation, or neuronal damage in HIE.

Together, these data support F3/flTF as an exploratory peripheral blood candidate marker for HIE. The evidence includes a nominal human whole-blood signal, directionally concordant rat cortex findings, PBMC upregulation in the clinical cohort, good discrimination in this cohort, and an association with disease severity. Large-scale validation, confounder-adjusted modeling, and mechanistic studies are needed before clinical translation.

## Conclusions

This study prioritizes F3, which encodes tissue factor, as an exploratory PBMC candidate marker for neonatal HIE. Public cross-species transcriptomic datasets were treated as separate biological resources. Rat cerebral cortex data provided orthology-mapped supportive context for F3 involvement in hypoxic-ischemic injury, but they were not merged with human whole-blood data and did not substitute for human blood validation.

Clinical validation showed that F3/flTF was upregulated at both the mRNA and protein levels in PBMCs from neonates with HIE. ROC analysis showed good diagnostic performance in this cohort, and elevated F3/flTF expression was associated with disease severity. These findings support further evaluation of F3/flTF as part of a multimarker blood-based strategy rather than as a stand-alone diagnostic marker.

Mechanistically, F3/flTF may reflect coagulation activation, vascular injury, endothelial dysfunction, and inflammatory responses during hypoxic-ischemic brain injury. Prospective multicenter studies are needed to validate diagnostic and prognostic utility, adjust for inflammatory and coagulation-related confounders, evaluate sampling-time effects, and clarify the functional role of F3/flTF in HIE.

## Data Availability

The original contributions presented in the study are included in the article/Supplementary Material, further inquiries can be directed to the corresponding author.
